# The Influence of the Printing Temperature and the Filament Color on the Dimensional Accuracy, Tensile Strength, and Friction Performance of FFF-Printed PLA Specimens

**DOI:** 10.3390/polym14101978

**Published:** 2022-05-12

**Authors:** Doina Frunzaverde, Vasile Cojocaru, Costel-Relu Ciubotariu, Calin-Octavian Miclosina, Deian Dorel Ardeljan, Emil Florin Ignat, Gabriela Marginean

**Affiliations:** 1 Department of Engineering Science, Babeș-Bolyai University, Traian Vuia Square 1–4, 320085 Reșița, Romania; doina.frunzaverde@ubbcluj.ro (D.F.); relu.ciubotariu@ubbcluj.ro (C.-R.C.); calin.miclosina@ubbcluj.ro (C.-O.M.); deian.ardeljan@stud.ubbcluj.ro (D.D.A.); e.ignat96@yahoo.com (E.F.I.); 2 Department of Materials Science and Testing, Westphalian University of Applied Sciences Gelsenkirchen Bocholt Recklinghausen, Neidenburgerstr. 43, 45897 Gelsenkirchen, Germany; gabriela.marginean@w-hs.de

**Keywords:** additive manufacturing, polylactic acid (PLA), fused filament fabrication (FFF), fused deposition modeling (FDM), printing temperature, filament color, dimensional accuracy, tensile strength, friction performance, wear

## Abstract

The printing variable least addressed in previous research aiming to reveal the effect of the FFF process parameters on the printed PLA part’s quality and properties is the filament color. Moreover, the color of the PLA, as well as its manufacturer, are rarely mentioned when the experimental conditions for the printing of the samples are described, although current existing data reveal that their influence on the final characteristics of the print should not be neglected. In order to point out the importance of this influential parameter, a natural and a black-colored PLA filament, produced by the same manufacturer, were selected. The dimensional accuracy, tensile strength, and friction properties of the samples were analyzed and compared for printing temperatures ranging from 200 °C up to 240 °C. The experimental results clearly showed different characteristics depending on the polymer color of samples printed under the same conditions. Therefore, the optimization of the FFF process parameters for the 3D-printing of PLA should always start with the proper selection of the type of the PLA material, regarding both its color and the fabricant.

## 1. Introduction

The Fourth Industrial Revolution, known as Industry 4.0, aims for the digital transformation of production processes and industries. Reaching one of the seven key themes of Industry 4.0, Digital Manufacturing and Design (DMD), additive manufacturing (AM) is considered to be part of the contemporary industrial revolution [1].

Additive manufacturing includes technologies that produce layer-upon-layer objects, hereby using a different fabrication principle than subtractive or formative production methods. Additionally called 3D printing, AM processes are based on three main types of software: Computer-Aided Design (CAD), Computer-Aided Manufacturing (CAM), and Firmware [2,3]. The 3D virtual model of the object is generated in CAD software and exported as an stl file. The printing process parameters and the infill properties are defined in the CAM (or slicer) software. The firmware software ensures the control and command of the 3D printer. Material profiles (sets of process parameters), predefined by the printer manufacturer or the filament producer, can be used in the printing process. In addition, the slicer software allows one to customize the printing variables according to the targeted properties of the printed part.

3D printing technologies were initially developed as a rapid prototyping tool for visualization and validation of the designed objects, as they allow the relative ease and cost-effective creation of complex geometries. Nowadays, the development of AM technologies has led from rapid prototyping to rapid manufacturing [4]. Polymers, metals, ceramics, and composite materials are used to fabricate industrial components [2], as well as products for the health sector [5,6,7], education purposes [8], leisure [2], and other fields.

Polymeric materials, including thermoplastics, thermosets, elastomers, functional polymers, polymer blends, and biological systems, account for the largest share of material classes used for 3D printing [9]. According to [2], seven process categories are applied for AM of polymers: binder jetting, directed energy deposition, material extrusion, material jetting, powder bed fusion, sheet lamination, and vat photopolymerisation. Among the corresponding technologies, one of the most widespread is the FFF (Fused Filament Fabrication) method, also known as the FDM (Fused Deposition Modeling) method [1,10].

Fused Filament Fabrication is an additive manufacturing technology that involves the extrusion of a molten thermoplastic filament through a moving printing head equipped with a nozzle of suitable diameter. The movement of the printing head is controlled by G codes, generated from the CAM software based on the CAD model and the process parameters, and the designed object is created by the overlapping of successive material layers, each realized by the deposition of multiple adjacent roads.

The FFF method has the advantages of low cost, regarding both equipment and suitable materials, commercially available in a broad diversity, the ease of use not only for industrial applications but also in laboratories and domestic environments, and the versatility of manufacturing products of different shapes and geometries, ranging from very simple to complex designs in a relatively short time [1,7,10].

On the other hand, creating functional parts for the end user by FDM proves to be a challenging task. The difficulties arise from the large number of process variables, which affect the part quality, in terms of dimensional accuracy, surface roughness, and staircase effect at curves, as well as its final characteristics. As for the latter, as pointed out by [11], one has to consider that the properties of the products obtained by FFF are anisotropic and strongly dependent on the part history and therefore are referred to as ”the strength of the part” instead of ”the strength of the part material”.

In this connection, when targeting good-quality FFF manufactured parts with specific properties, one should take into consideration at least the following influential printing process variables [12,13]:

− The type of the filament (material, color, diameter, producer) and the storage conditions before the printing process (environmental humidity, UV-irradiation).
− The model design and the infill parameters (infill pattern, infill density).
− The process parameters (layer thickness, printing speed, printing temperature, build plate temperature, number of identical specimens printed at once, build orientation, raster angle).
− The type of 3D printing equipment (producer, open or closed workspace).
− Post-process treatments, storage conditions after the printing process, and aging.
− The test methods and the test parameters applied.

The development and optimization of the process parameters, in order to obtain FDM prints with good surface quality, dimensional accuracy, and predictable properties, is a topic that has been addressed over many years by an impressive number of researchers. Experimental investigations, as well as statistical methods, such as the design of experiments (DOE), the Taguchi method, and an analysis of variance (ANOVA), were applied, aiming to define the optimal combinations of the printing variables that could ensure the best quality and the desired properties of the FFF-printed samples [10,14].

The most commonly used filament material for FDM printing is Poly-Lactic Acid (PLA). PLA is a biodegradable, thermoplastic, and semi-crystalline polymer, which is preferred by mainstream users over other filament materials because of its lower melting point (150–160 °C), enabling printing on less expensive equipment, as well as its lower toxicity in comparison to other thermoplastics (such as ABS), if degraded in the printing process [15]. A very important characteristic of PLA, which makes it suitable for use in medical applications, is its biocompatibility [16]. Sometimes, the intrinsic properties of pure PLA cannot ensure certain properties needed for specific products, such as, for example, products with high electrical and thermal conductivity. For the improvement of such properties, additives are mixed with PLA, leading to PLA-based composites [16,17,18,19].

The printing temperature (also referred to as the printing head temperature or the extrusion temperature) and its influence on the FDM-printed part’s quality and mechanical properties is one of the most studied process parameters. In this regard, researchers tested temperatures ranging from 175 °C to 275 °C, but the most commonly analyzed temperatures were situated between 190 °C and 220 °C [12].

The extrusion temperature affects the dimensional accuracy of the FDM-printed PLA objects. Dimensional accuracy is essential regarding the equipment’s reliability to produce parts according to the expected results and the fit into designed structures [20]. Higher temperatures determine an increase in the filament fluidity, allowing it to expand more freely and thereby causing larger dimensional deviations [1,4,21,22]. As pointed out in [23], FDM manufacturing currently leads to PLA parts with larger dimensions as compared to the CAD model.

According to previous research, the effect of the printing temperature on the tensile strength of PLA specimens printed by FFF is determined both by the part mesostructure and the crystallinity content of the semi-crystalline PLA polymer.

Regarding the influence of the printing temperature on the mesostructure and its effect on the part’s mechanical properties, it is unanimously accepted that too-low temperatures cause under-extrusion defects (delamination of successive layers, larger gaps between raster lines) and thereby lower tensile strength. On the other hand, too-high selected printing temperatures lead to negative effects of over-extrusion, consisting of voids caused by filaments with too-low viscosity (bubbles determined by unsteady flow from the nozzle, layer drooping, stringing, oozing), also diminishing the tensile strength [15,21,24]. The selection of the proper printing temperature, avoiding under- and over-extrusion, has a positive effect on the part’s compactness and thereby on its tensile strength [4,7,11,13,22,23,25,26].

As a consequence of those presented above, considering both the dimensional accuracy and tensile strength, some researchers [23] concluded that it may not be possible to maximize the mechanical properties and minimize the dimensional errors at the same time through only the selection of a proper printing temperature. For this purpose, multi-objective optimization should be applied by taking into consideration the combined effect of the extrusion temperature and other printing parameters.

Concerning the influence of the FDM extrusion temperature on the crystallinity of PLA-printed parts and, through this, on its mechanical properties, controversial opinions are stated in previous studies. Some researchers [27,28,29] reported a strong and color-dependent relationship between the printed sample’s crystallinity and the extruder temperature, and that improper temperature control reduces the crystallinity of the 3D print [30]. Moreover, the influence of the printing temperature on the in-process crystallinity does not show linear variation, and there is a critical temperature for each type of FDM-printed PLA filament at which a maximum percent crystallinity can be achieved [27]. Contrarily, other researchers [22] came to the conclusion that the variation of the printing temperature does not have a significant effect on the crystallinity of the PLA samples or that, although the in-process crystallinity of the printed PLA varied between 5.3 and 19.6%, related to the 10% crystallinity of the as-received yellow filament, no evident correlation between the increase in the tensile strength and the crystallinity obtained during the FDM process can be established, because of the low level of in-process crystallinity. As a consequence, the quality of the mesostructure is considered to play the primary role in achieving tensile strength [31]. Contrariwise, other authors [32,33] reported that the materials with higher in-process crystallinity show correspondingly increased mechanical properties.

The abovementioned opinion dissimilarities regarding the in-process variation of the crystallinity in the dependence on the printing temperature and the related effect on the part strength may be a consequence of the fact that the investigations were carried out on PLA samples manufactured with different process parameters. In addition, most of the authors do not publish complete information regarding the type of the PLA filament used, diameter, color, and filament producer, and these characteristics of the material may influence the printed part characteristics.

Regarding the color of the PLA filament, previous research showed that its influence on the dimensional accuracy [1,34] and the tensile strength [17,18,27,29] of FDM-printed samples cannot be neglected, and that also the same color PLA filaments manufactured by different producers, may not have the same printing properties [24]. The reasons behind this are considered to be the color-dependent in-process crystallinity and/or the different thermal behavior [17,34] in the case of PLA filaments containing coloring additives. Some researchers also more precisely explained the reasons for the abovementioned effects, indicating that the coloring agents may act as crystallization rate modifiers [27] or nucleation agents [19] and may restrict the material flow during the printing process [27].

With respect to the friction and wear performance of PLA samples printed by FDM, the authors of previous research pointed out the fact that both parameters, the color of the PLA filament [35] and the extrusion temperature [36,37], influence these characteristics. In relation to the friction performance, the surface finishing of the parts has to be considered and therefore the influence of the filament color [29,34] and the extrusion temperature [22,34] on the sample’s roughness. Some of the researchers also reported that natural PLA prints exhibited the lowest roughness value compared to that of other colored PLA filaments, manufactured under the same process conditions [1]. Furthermore, the negative effect of black color additives on the wear resistance of FDM-printed PLA specimens was reported [35]. However, it may be observed that few authors have addressed this topic in relation to the impressive number of researchers that have analyzed the influence of the same parameters on the mechanical behavior of FDM-printed PLA parts.

The main objective of the present work was to realize a comparative study regarding the influence of the printing temperature on the dimensional accuracy, tensile strength, and friction performance of two types of PLA filaments, produced by the same manufacturer but having different colors (natural and black). In order to exclusively point out the effect of the filament color and the temperature on the quality and the targeted properties of the FDM samples, all other printing parameters were fixed.

## 2. Materials and Methods

The analysis regarding the influence of the printing temperature and the filament color on the dimensional accuracy, mechanical properties, and friction performance of FFF-printed PLA was carried out on two types of specimens, as follows:

− Prismatic specimens (length a_1_ = 30 mm, width a_2_ = 30 mm, and height h = 10 mm) for the evaluation of the dimensional accuracy and friction performance.
− ISO 527-2:2012 type 1A [38] tensile specimens for mechanical testing.

The FFF printing of the specimens was performed on a Creality Ender (Creality, Shenzhen, China) printer with Marlin firmware. The 3D printer has a print volume of 12.1 L and a working platform of 220 mm/220 mm/250 mm. Brass nozzles 0.4 mm in diameter were used in the printing process. The maximum temperature of the print head on this printer is 255 °C and the maximum temperature of the build plate is 110 °C. The 3D models of the specimens were designed with SolidWorks (Dassault Systèmes, Vélizy-Villacoublay, France) and the G-code files were generated with Ultimaker Cura 4.12 (Ultimaker B.V., Utrecht, The Netherlands).

Aiming to point out the color-dependent behavior of the 3D print, two different types of filament were used: Verbatim PLA Filament 1.75 mm—Natural; and Verbatim PLA Filament 1.75 mm—Black (CMC Magnetics Corporation, Taipei, Taiwan). The printing parameters that were applied for the specimens are shown in Table 1. All the samples were individually printed.

The fixed parameters were as follows: the layer thickness (0.2 mm), targeting both acceptable values for the ultimate tensile strength (UTS) and the printing duration [12]; the build plate temperature (60 °C), as recommended by the filament producer; the printing speed (50 mm/s), higher than the value mentioned by the filament producer, in order to reduce the printing time of the samples and also considering previous research [12,23,24,26,31]; the build orientation (YX) and the raster angle (45°/−45°) for better mechanical properties [12]; and the infill density (100%), in order to minimize the influence of this parameter on the printed samples’ mesostructure.

The variable parameters were the printing temperature (ranging from 200 °C to 240 °C) and the filament color (natural PLA and black PLA). The natural PLA filament was selected as the reference material, whereas the black-colored PLA was chosen considering the fact that black dyes enhance the thermal conductivity of the PLA and modify its friction behavior, as shown by previous research [3,17,18,28,29,34,35].

For the evaluation of the dimensional accuracy and the tensile test, five specimens were printed for each color–temperature combination. The friction behavior was determined on two prismatic samples for each combination, selected from the series used previously for the measurements of the dimensional deviations of the 3D prints.

The dimensions of the 30/30/10 samples were measured by means of a digital caliper (accuracy 0.01 mm), considering three values for each main specimen dimension, determined at different measuring points on a_1_ = 30 mm, a_2_ = 30 mm, and h = 10 mm. Five specimens were evaluated for each color–temperature combination (25 PLA black specimens and 25 PLA natural specimens). For each prismatic specimen, the effective volume was calculated using the average values resulting for a_1_, a_2_, and h. The average volumes and the standard deviations (calculated according to ISO 2602:1980 [40]) were represented graphically for each color–temperature batch. The confidence level for the mean was 95%.

The tensile tests were performed according to ISO 527-1:2019 [41] and ISO 527-2:2012 [38], at a test speed of s_p_ = 10 mm/s , on a Mecmesin MultiTest 2.5-dV (PPT Group UK Ltd, Slinfold, United Kingdom) testing machine equipped with an ELS-S 2500 N force cell. Mecmesin’s Vector Pro MT software was used for the test control and the data acquisition. Five tensile specimens (25 PLA black specimens and 25 PLA natural specimens) were tested for each color–temperature combination and the average value of the ultimate tensile strength (UTS) was calculated. The average UTS and the standard deviations (calculated according to ISO 2602:1980 [40]) were represented graphically for each color–temperature combination. The confidence level for the mean was 95%.

In order to evaluate the mesostructure of the FDM prints with respect to their color and temperature dependence, both the surface and the fracture surface of the tensile test specimens were examined by a Leica MZ 7.5 stereomicroscope (Leica Microsystems, Wetzlar, Germany) at a magnification of 10×.

The friction behavior of the FDM-printed specimens was investigated by the pin-on-disc test, performed on a CSM tribometer (CSM Instruments Tribometer, Massachusetts, USA) in accordance with ISO 7148-2:2012(E) [42]. The following test parameters were applied: counterpart—Al_2_O_3_ ball, with 6 mm diameter; wear track radius—7 mm; normal load—10 N; sliding speed—150 mm/s; temperature—23 °C; humidity—50%. The tests were performed for up to 10,000 laps on two specimens for each color–temperature combination. The variation of the friction coefficient during the test was recorded for all samples, as well as the corresponding mean value. For the comparative analyses of the obtained results, the average value of the friction coefficients for each set of two specimens of the same color printed at the same temperature was calculated, using the mean values given by the software of the tribometer for each tested sample.

The wear profile of the natural PLA and black PLA specimens printed at 210 °C was evaluated using a Keyence confocal laser scanning microscope—CLSM (Keyence VKX-260K, Neu-Isenburg, Germany).

## 3. Results and Discussions

The experimental results pointing out the influence of the printing temperature and the filament color on the dimensional accuracy, mechanical properties, and friction performance of FFF-printed PLA are presented and discussed in the following subsections. For the ease of visualization, in all the figures representing the comparative values of the dimensions, for the properties (ultimate tensile strength and friction coefficient) of the natural and the black PLA filaments, the same two colors were selected, as follows: yellow for natural PLA and grey for black PLA.

### 3.1. Dimensional Accuracy

**Figure 1:**
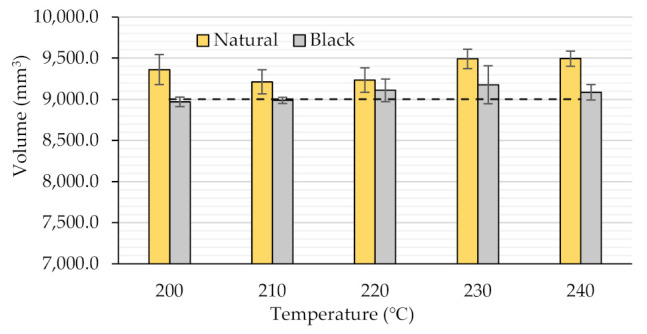
The variation of the volume (prismatic specimen with theoretical volume: 9000 mm^3^, dashed line) with the printing temperature and the filament color.

As the authors had the intention to analyze the temperature and color dependency of the dimensional accuracy, without also considering the influence of the build orientation or the raster angle, in order to point out the overall effect of the two varied parameters on the samples’ dimensions, the effective volume of the prismatic specimens, determined as described in Section 2, was taken into consideration. The corresponding results, clearly visualizing the sense and magnitude of the absolute volume deviations in relation to the model’s theoretical volume, are presented in Figure 1.

As revealed in Figure 1, the extrusion temperature has a significant effect on the dimensional accuracy of the FDM-printed specimens. When the extrusion temperature increases, the overall tendency in the case of both types of PLA filaments shows that the dimensional errors increase. This might be explained by the increased fluidity of the extruded materials at higher temperatures, which allows the filaments to expand freely and hereby makes dimensional control more difficult, as also mentioned by other authors [4,21,22]. Regarding Figure 1, one may also observe that, for temperatures ranging between 200 °C and 220 °C in the case of the natural PLA and beginning with 230 °C for the black PLA, the dimensional deviations of both filament types slightly differ from the general trend, which could be a consequence of the in-process intrinsic structural changes of the semi-crystalline polymers and their corresponding properties, as will be further discussed in Section 3.2.

The experimental results also showed that, in agreement with the considerations expressed in [23], the FDM fabrication process usually generates parts with larger dimensions as compared to the CAD model.

As for the influence of the filament color on the dimensional accuracy, Figure 1 clearly reveals that this parameter has to be taken into consideration when aiming to obtain parts with precise dimensions. The best overall dimensional accuracies were obtained in the case of the black PLA filament, where the maximum volumetric deviation reached 1.96% (at 230 °C) in relation to the model’s volume, compared to 5.50% (at 240 °C) in the case of the natural PLA. This conclusion agrees with the results presented in [20], where the best overall dimensional accuracies, in comparison to white and grey filaments, were obtained for black PLA.

### 3.2. Tensile Behavior

**Figure 2:**
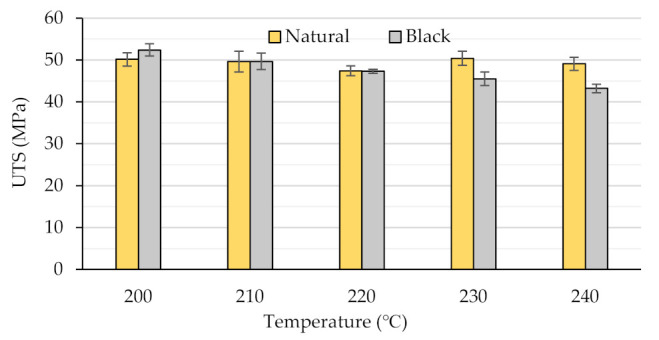
The variation of the ultimate tensile strength (UTS) with the printing temperature and the filament color.

The values of the ultimate tensile strength (UTS), determined for the natural and the black specimens, printed at temperatures ranging from 200 °C to 240 °C, are presented in Figure 2. As described already above, a set of five tensile specimens were tested for each color–temperature combination.

The experimental results with respect to the overall values and the temperature dependence of the UTS determined for the different colored tensile samples clearly proved that coloring agents added to the PLA material influence its mechanical behavior.

Aiming to correlate these results with the samples’ mesostructures, the surfaces of the tensile specimens, as well as their fracture surfaces after tensile testing, were examined by light microscopy, as described in Section 2. Representative images for each set of color–temperature combinations are shown in the following figures.

**Figure 3:**
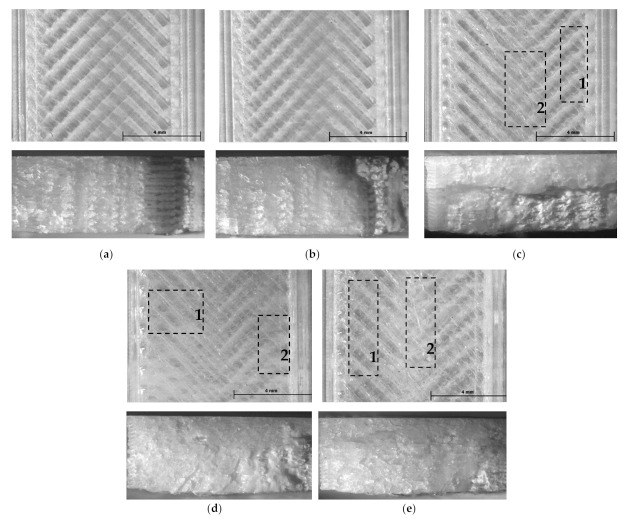
Top view (up) and fractured surface (down) of natural PLA tensile specimens printed at (**a**) 200 °C, (**b**) 210 °C, (**c**) 220 °C, (**d**) 230 °C, (**e**) 240 °C.

Both the surface and the fracture surface of the tensile specimens made of natural PLA (Figure 3) obviously show that the compactness of the structures was improved by increasing the printing temperatures due to the continuous increase in the fluidity of the material. Above 220 °C, this phenomenon led to the unsteady flow of the molten natural PLA filament and over-extrusion structural defects (non-uniform filament roads, marked by 1, and oozing, marked by 2, Figure 3c–e). Contrarily, in relation to the structural aspects, the minimum value of the UTS was reached by the tensile samples printed at 220 °C (UTS = 47.43 MPa) and the highest UTS value was obtained for the extrusion temperature of 230 °C (UTS = 50.41 MPa). Admitting that the tensile strength of FDM-printed samples is dependent on the degree of the print’s crystallinity, as demonstrated by [7,27,43], this non-linear variation of the UTS values may be explained by the temperature-dependent in-process modification of the samples’ crystallinity, that, for the given printing conditions, seems to play the leading role regarding the mechanical behavior of the natural PLA specimens. As pointed out by [27], there appears to be an optimal extrusion temperature for each color of the PLA filament to optimize the degree of crystallinity.

Regarding the black PLA, as shown in Figure 2, the UTS values varied between 52.41 MPa and 43.23 MPa and were continuously decreasing as the printing temperature increased from 200 °C to 240 °C. Compared to the natural PLA prints, for temperatures between 200 °C and 210 °C, the black samples showed higher UTS values. This was not the case upon a further increase in the printing temperature, as the UTS of the black-colored specimens continued to decrease, while that of the natural PLA samples increased. Low values of the UTS of black PLA were also reported by other researchers [17,18,29] for temperatures situated in the same range. Considering the fact that coloring agents added to natural PLA may act as nucleating agents for the crystalline regions of the semi-crystalline polymers, as previously demonstrated by other authors [19,27], one might assume that the printed black PLA has higher crystallinity than that of the printed natural PLA. The higher degree of crystallinity led not only to higher UTS values for the initial printing temperatures (200 °C to 210 °C), but also assured a higher melting point of the black filament, in accordance with [19,33]. The higher values of the melting temperature, as well as of the higher thermal conductivity of the black filler, pointed out by [17,18], caused a completely different thermal behavior of the black filament in comparison to the natural one for the printing temperatures situated between 200 °C and 240 °C. The influence of the coloring additives on the thermal behavior during the printing of PLA was also reported in [34].

**Figure 4:**
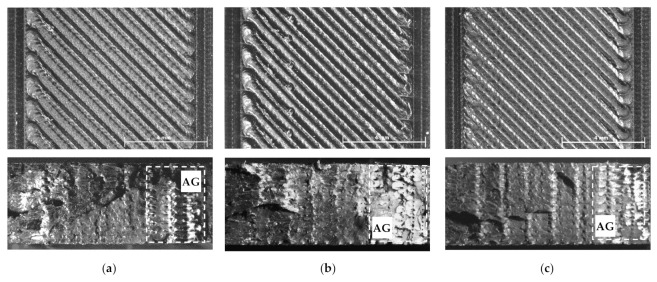

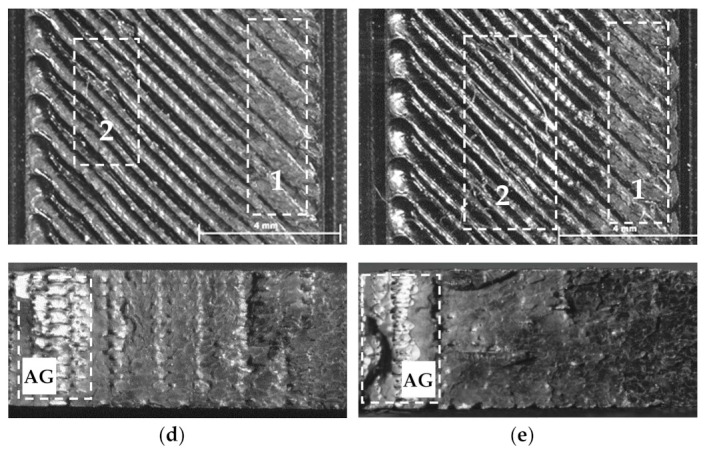
Top view (up) and fractured surface (down) of black PLA tensile specimens printed at (**a**) 200 °C, (**b**) 210 °C, (**c**) 220 °C, (**d**) 230 °C, (**e**) 240 °C.

As a consequence of the abovementioned factors and as revealed by Figure 4a–e, air gaps (marked by AG) between the printed roads and the successive layers occurred in the mesostructure of all black specimens manufactured up to 240 °C. This can be visualized particularly at the turn of the roads, near the contour wall, where the cooling rate of the material was higher due to the enhanced heat exchange with the environment. These observations are in accordance with [44], where it was found that similar gaps increased when the printing temperatures were situated in the range of 190 °C to 230 °C. Their occurrence may be related to the fact that the black conductive filament solidified and contracted differently at higher temperatures than the natural PLA and the related shrinkage of the material produced enough force to overcome the adhesion between the already-deposited roads. The air gaps likely initiated the fracture at lower UTS values for the black PLA samples in comparison with the natural PLA ones. It determined the continuous loss in tensile strength while increasing the extrusion temperature as well. Over-extrusion effects, such as non-uniform filament roads (marked by 1) and oozing (marked by 2), were also observed on the surface of the black tensile samples printed at temperatures higher than 230 °C (Figure 4d,e).

### 3.3. Wear

**Figure 5:**
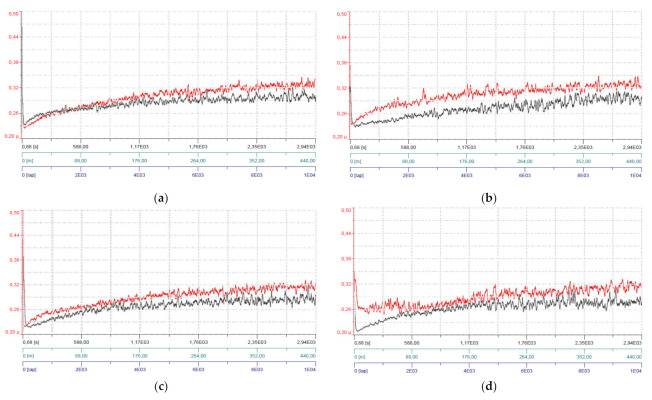

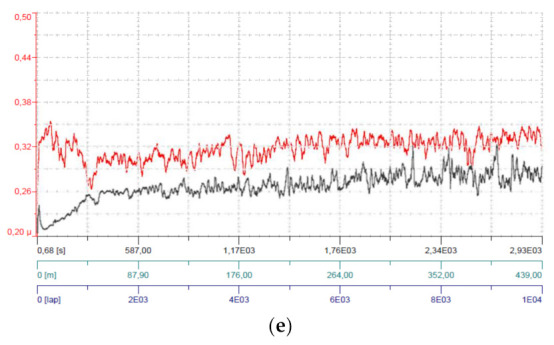
Evolution of the friction coefficient for natural (red curves) and black (black curves) PLA during tribological testing: (**a**) 200 °C (**b**) 210 °C (**c**) 220 °C (**d**) 230 °C, (**e**) 240 °C. Axis explanations: Vertical axis: μ (friction coefficient); Horizontal axis: [s] (test duration, up to 2930 s), [m] (sliding distance, up to 439 m) and [lap] (number of laps, up to 10,000 laps).

The results obtained from the tribological tests of the PLA samples produced under the same parameters were very similar, therefore Figure 5 shows the variation of the friction coefficients during the total test duration just for one sample made of natural PLA and black PLA, printed at temperatures ranging from 200 °C to 240 °C.

The natural PLA samples presented, for all printing temperatures, higher friction coefficients than that of the black PLA specimens. This fact may be explained by the different surface finishing of the natural and black PLA samples (Figure 3 and Figure 4), as the natural specimens’ surfaces were visibly smoother for all extruder temperatures than those of the black prints. Finer surface finishing results in enhanced sliding contact between the test piece and the counterpart and thereby more intense friction processes. On the contrary, rougher surface finishing is conducive to less contact between the two parts of the tribological system and, through this, lower friction.

Regarding the stabilization of the friction process, one can observe that the steady-state was reached, for all temperatures, almost at the same time for both color prints, as follows: a—3000 lap; b—2000 laps; c—3000 laps; d—2000 laps; e—1000 laps.

**Figure 6:**
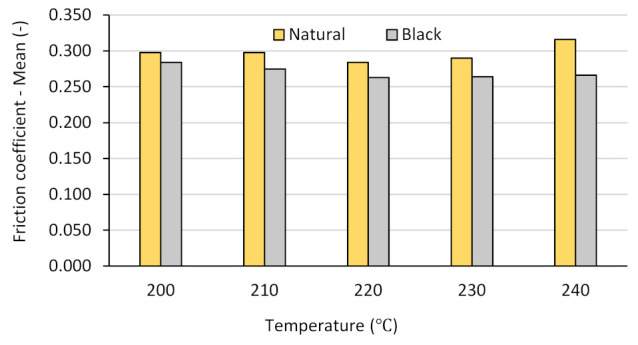
The variation of the mean value of the friction coefficient with the printing temperature and the filament color.

Aiming to also visualize the tendency of the temperature-dependent variation of the friction coefficient for both filament colors, the mean values of the registered friction coefficients are represented in Figure 6. The values are in accordance with the values mentioned in previous research work [30] for FDM-printed PLA tested under similar conditions.

As shown in Figure 6, the variation of the mean value of the friction coefficients dependent on the printing temperature is interesting, following, for both colors, the same variation tendency as that of the UTS (Figure 2). The reasons for the different tribological behavior of the natural and black samples might be explained by the different properties and structures of the samples.

Thus, one can observe that, in the case of the of natural PLA samples, the friction coefficients manifested an overall increasing tendency as the printing temperature varied from 200 °C to 240 °C, corresponding to the gradually increasing fluidity of the filament that determined both a more compact mesostructure of the samples and a finer surface finishing (Figure 3a–e). The decrease observed around 220 °C was likely a consequence of the lower crystallinity of the natural PLA, as amorphous polymers are soft and may suffer deformations during the friction process [27,33,41], resulting in a decreased contact surface between the tested part and the counterpart. This phenomenon is pointed out below.

As for the black samples, the continuous decrease in the friction coefficient with the increase in the printing temperature was determined by the smaller area of friction contact due to the deterioration of the samples’ surface finishing (Figure 4a–e), which, in turn, was a consequence of the increased fluidity of the conductive black PLA filament, its higher melting temperature, and the solidification of the deposited roads without proper binding, as already discussed in Section 3.2. The poor surface quality of black PLA samples printed at 225 °C was reported also by [29].

The wear tracks of the natural and black PLA specimens printed at 210 °C were investigated by digital microscopy to better evaluate the overall wear behavior. This extruder temperature was chosen regarding the acceptable dimensional accuracy and the tensile strength values exhibited for both types of PLA filament colors. The recorded images are presented in the following figures.

**Figure 7:**
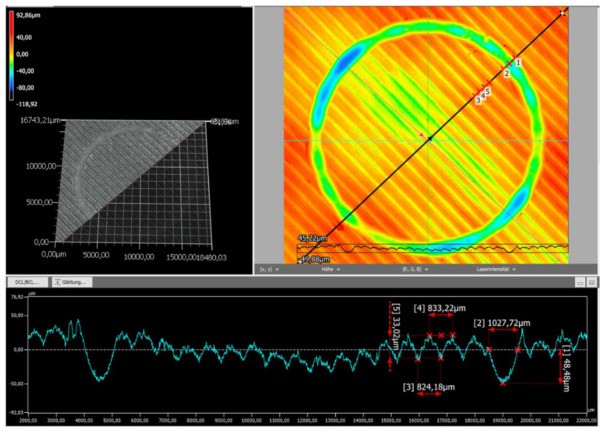
The wear profile of the natural PLA test specimen printed at 210 °C.

**Figure 8:**
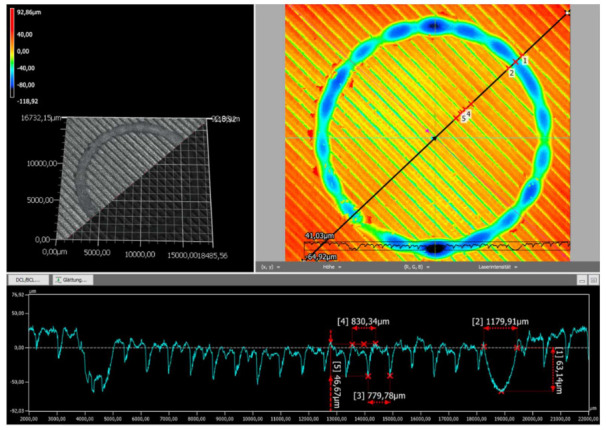
The wear profile of the black PLA test specimen printed at 210 °C.

As revealed in Figure 7, compared to Figure 8, the surface finishing of the natural PLA specimen was smoother than that of the black sample, but rather deformed. The generated wear track of the natural PLA sample exhibited a non-uniform depth distribution with values up to 80.00 μm (see Figure 7). The exemplary dimensions of the wear track in the registered direction (along the black line, Figure 7) are a width of 1027.72 μm and a depth of 48.48 μm.

Figure 8 shows the surface finishing and the wear track profile of the black PLA sample in direction 2. It may be observed that the surface of the black specimen was much rougher than that of the natural color print, as already revealed by Figure 3 and Figure 4 displayed above. The wear track of the black PLA sample was also non-uniform, but its overall depth was higher than that of the natural PLA sample, reaching the value of 118.92 μm. Exemplar wear track dimensions in the registered direction (along the black line, Figure 8) were a width of 1179.91 μm and a depth of 63.14 μm. The maximum wear depth associated with the coarse surface finishing of FDM-printed black PLA was also reported by [35].

Regarding the profiles of the wear tracks of the natural and black PLA samples, the different wear behavior of the specimens produced from the two types of PLA filaments could again be a consequence of the different in-process percent crystallinities achieved by the samples during printing and their corresponding mechanical and thermal behavior. In this regard, as presented in [33], polymers with a high degree of crystallinity are rigid and have a high melting point, while amorphous polymers are soft and have lower melting points. Thus, the higher depth of the black material’s wear track indicates that the wear process was mainly abrasive, and the wear debris was removed from the sample’s surface during the test. In the case of the natural PLA, the lower depth of the track resulted from adhesive processes as the heat developed due to the contact friction conducive to the formation of transfer layers.

## 4. Conclusions

The optimization of the process parameters for the production of FFF-printed PLA parts with good surface quality, dimensional accuracy, and suitable properties is a topic that has been addressed for many years by an impressive number of researchers. Experimental investigations, as well as statistical methods, were applied, aiming to define the optimal combinations of the printing variables that could ensure the best quality and desired properties of the FFF-printed samples.

In this respect, the filament color is the least-addressed factor in previous research. Moreover, the color of the PLA filament, as well as its manufacturer, are rarely mentioned together with the experimental 3D printing parameters, although current existing data reveal that their influence on the final characteristics of the prints should not be neglected.

The present work reports two types of PLA filament: Verbatim PLA Filament 1.75 mm—Natural, and Verbatim PLA Filament 1.75 mm—Black. We aimed to reveal the influence of the color on the characteristics of the FFF-printed PLA materials in correlation to the dimensional accuracy, the tensile strength and the friction properties of the samples. Therefore, the printing temperature was the only process variable considered, as its influence on FFF-manufactured objects’ quality and properties had been demonstrated by previous research.

Based on the results obtained from the PLA samples, the optimal characteristics with respect to the dimensional accuracy, tensile strength, and sliding wear behavior were identified in the temperature range of 210 °C to 220 °C for the natural PLA and at slightly lower temperatures in case of the black PLA (200–210 °C). Both types of PLA, printed at the upper limit of the previously mentioned temperature ranges, exhibited lower values of the UTS and friction coefficient. The samples printed above 220 °C (natural PLA) and 210 °C (black PLA) showed degradation of surface quality and mesostructures, a fact that is in accordance with their determined properties.

Considering the abovementioned results, an important conclusion to draw is that the optimization of the FFF process parameters for the 3D-printing of PLA, in order to obtain the best combinations for end-user products’ quality and properties, should always start with the proper selection of the type of the PLA material, regarding both its color and the fabricant. Therefore, further investigations on the influence of coloring additives on the in-process modifications of the structure (crystallinity of the semi-crystalline PLA polymers, degradation) and the physical properties of the materials have to be carried out by researchers, preferably in collaboration with PLA filament producers, so that these modifications can be known and thereby controllable.

## Figures and Tables

**Table 1:** 3D-printing parameters.

Parameters		Values
Fixed process parameters	Layer thickness, t	0.2 mm
	Printing speed, sp	50 mm/s
	Build plate temperature, TB	60 °C
	Nozzle diameter, dn	0.40 mm
	Filament diameter, df	1.75 mm
	Build orientation (acc. to [39])	YX
	Raster angle, θ	45°/−45°
	Infill density	100 %
	Number of wall lines, WL (-)	2
Variable parameters	Printing head temperature, TH	200 °C; 210 °C; 220 °C; 230 °C; 240 °C
	Material/Filament color	PLA Natural; PLA Black

## Data Availability

The data presented in this study are available on request from the corresponding author.
